# HUIL-TN & HUI-TN: Mining high utility itemsets based on pattern-growth

**DOI:** 10.1371/journal.pone.0248349

**Published:** 2021-03-12

**Authors:** Le Wang, Shui Wang

**Affiliations:** College of Digital Technology and Engineering, Ningbo University of Finance and Economics, Ningbo, Zhejiang, China; Sejong University, KOREA, REPUBLIC OF

## Abstract

In recent years, high utility itemsets (HUIs) mining has been an active research topic in data mining. In this study, we propose two efficient pattern-growth based HUI mining algorithms, called High Utility Itemset based on Length and Tail-Node tree (HUIL-TN) and High Utility Itemset based on Tail-Node tree (HUI-TN). These two algorithms avoid the time-consuming candidate generation stage and the need of scanning the original dataset multiple times for exact utility values. A novel tree structure, named tail-node tree (TN-tree) is proposed as a key element of our algorithms to maintain complete utililty-information of existing itemsets of a dataset. The performance of HUIL-TN and HUI-TN was evaluated against state-of-the-art reference methods on various datasets. Experimental results showed that our algorithms exceed or close to the best performance on all datasets in terms of running time, while other algorithms can only excel in certain types of dataset. Scalability tests were also performed and our algorithms obtained the flattest curves among all competitors.

## 1 Introduction

Pattern discovery has been an important topic in data mining and has been applied in many fields [1–4]. Since the development of the Apriori algorithm for mining frequent itemsets [1], new algorithms [2, 5] have been continually proposed for various formulations and performance enhancements. Traditional frequent itemset mining treats each item in a transaction as binary. In other words, each itemset either occurs or does not occur in a transaction. However, in the real-world, real-valued quantities, such as profit, may be practically important. The unit profit and purchase quantity of items are vital for finding the most valuable itemsets that contribute most to the overall profit. To address this problem, mining HUIs (High Utility Itemsets) was proposed and studied extensively in the data mining literatures [6–11].

A typical method of mining HUIs consists of two steps: generating candidates and calculating utility values of these candidate itemsets. The performance bottleneck of these algorithms has primarily been in the candidate generation process, thus lots of research efforts have been focused on improving this step by reducing the number of candidates or avoiding the candidate generation step completely.

For example, a number of algorithms that generate candidate itemsets based on the apriori method [12–14] or map the transaction itemsets to utility lists [8, 15] may generate non-existing candidate itemsets (i.e., itemsets that may co-occur but never co-occurred in the dataset), which results in unnecessary computing that negatively impacts the computational performance.

In contrast, pattern-growth based algorithms [10, 16, 17] can avoid generating non-existing candidates, thus are promising for superior computation performance in HUI mining. Although the pattern-growth approach can effectively exclude non-existing itemsets from candidate itemsets, they still generate candidate itemsets and require additional scans of the original dataset to calculate the exact utility value of these candidates and identify HUIs. The reason is that they apply an *over-estimated utility value* to generate candidate itemsets after mapping transaction itemsets to a tree structure. Unlike in frequent pattern mining, the downward closure property of the support measure is no longer applicable in HUI mining, and cannot be used for effectively removing low utility patterns from the candidates. Therefore, an over-estimated utility value that has a computation friendly downward closure property has been a commonly adopted strategy in HUI mining [13, 14, 18–21]. The bigger the over-estimated utility value, the more candidates the algorithm will generate, and thus the less efficient it will be. Without the ability of directly retrieving the exact utility values from the tree, existing pattern-growth based HUI mining methods need to scan the original dataset to identify HUIs, which required additional passes of data I/O, resulting in much computation overhead.

There is a fact that the *maxlength* of HUIs is less than the length of many transactions in many datasets, so we can reduce the number of items in global header table and sub-header table if we calculate TWU value of items using part items of transactions, not all items of transactions.

Bearing in mind the above reasoning, we propose a novel tree structure, called tail-node tree (TN-tree), from which we can retrieve the exact utility value of an exisiting itemset without re-scanning the original dataset. The basic idea is that we maintain the utility of each individual item in the itemset in a special node (called the *tail-node*) when mapping the transaction itemsets to a tree. Correspondingly, the *tail-node tree* based HUI mining algorithms, named HUIL-TN and HUI-TN, are proposed for discovering HUIs efficiently. With this concise enhancement, our algorithms can find HUIs from a dataset with outstanding performance. Experimental results with both dense and sparse datasets also verified the effectiveness of the proposed methods.

Our contributions may be summarized as follows:

1. We designed a novel tree structure whose tail-nodes are used to store item-specific utility information, so that the exact utility value of an itemset may be easily retrieved later.
2. Based on the pattern-growth approach, we designed two HUI mining algorithms with no need of generating candidate itemsets: one uses part items of transactions and another one uses all items of transactions when creating global header table and sub-header tables.
3. Extensive experiments under different situations were performed to compare HUIL-TN and HUI-TN with four state-of-the-art algorithms EFIM [7], D2HUP [8], HMiner [6] and ULBMiner [9]. Experimental results demonstrated that the proposed algorithms outperformed the state-of-the-art algorithms.

The rest of this paper is organized as follows. section 2 describes related work for HUI mining. section 3 describes the background. section 4 describes the proposed algorithms. section 5 reports our experimental results. section 6 draws the conclusions and point out possible future work.

## 2 Related work

Existing HUI mining algorithms may be categorized into two groups: two phase algorithms and one phase algorithms.

### 2.1 Two phase algorithms

Yao et al. proposed the mathematical model for mining HUIs [14]. The authors estimated an expected utility value to determine whether an itemset should be a candidate itemset for high utility itemsets. However, the number of candidates may approach the number of all the combinations of items if the minimum utility value is very small and a dataset contains many distinct items, so the mining process might be time-consuming. Later, Yao et al. proposed two new algorithms for mining HUIs: UMining and UMining_H [21]. Algorithm UMining employs the utility upper bound property for pruning. UMining_H employs a heuristic method for pruning. These two algorithms may prune some HUIs, and also suffer from excessive candidates.

Liu et al. proposed algorithm Two-Phase [13] for mining HUIs. The authors firstly proposed the *Transaction-Weighted-Utilization* (TWU) model. The model maintains a TWU downward closure property. In this model, an itemset can be considered as a candidate itemset for HUIs if its TWU value is not less than a minimum utility value. Two-Phase consists of two phases; in the first phase, Two-Phase finds all the candidate itemsets; in the second phase, the algorithm discovers the actual HUIs from the candidate itemsets by an additional dataset scan. This algorithm outperforms the algorithm proposed in the paper [14]. However, this algorithm still generates too many candidates in the first phase and needs multiple scans of the dataset.

To reduce the number of candidates in the first phase of algorithm Two-Phase, Li et al. proposed an isolated items discarding strategy (IIDS) to reduce the number of candidates and applied the strategy to the two existing algorithms, and get two new algorithms renamed FUM and DCG+ [12]. These two new algorithms outperformed their original predecessors. Although IIDS effectively reduces candidates, it still scans dataset multiple times and generates candidate itemsets for HUIs.

In order to avoid multiple scans of the dataset for algorithms based on Pattern-growth, HUP-Growth [19] creates HUP-Tree in a way like FP-Tree. When mapping a transaction itemset to a tree, it stores the utility values of this node as well as the node’s ancestors into a list (this list is called “utility list”). If the node’s utility list already exists, the itemset’s utility values are added up to the list. This way the utility values of all possible itemsets of the dataset can be calculated from the tree. HUP-Growth takes a bottom-up approach to process each item, collecting items along the path, generates all possible combinations containing this item, and calculates their utility values, thereby determines all HUIs for current item. The merit of this algorithm is that utility values of itemsets can be calculated efficiently from the tree. But it still generates too many candidate itemsets.

Algorithm IHUP [16] also adopts FP-Tree’s approach to create IHUP-Tree. When it maps a transaction itemset to a tree, the utility value of this transaction itemset is stored on each node of this itemset. If the node already contains a utility value, new value is simply added to it. IHUP utilizes pattern-growth approach (FP-Growth method [2]) to generate candidate itemset, and uses the sum of all utility values of the corresponding nodes of the current item as the over-estimated threshold to determine whether this itemset is a promising candidate. Compared with HUP-Growth’s approach that combines items along the path to get candidate itemsets, IHUP’s candidates are lesser and the mining efficiency is increased.

IHUP cannot retrieve an itemset’s utility value directly after it maps the transaction itemset to a tree. Instead, it gets the sum of all utility values of the transactions containing this itemset (over-estimated utility value). Therefore, it needs to scan the original dataset to calculate candidates’ utility after these candidates are generated. Algorithm UP-Growth [10] is an improvement of IHUP. When it maps a transaction itemset to a tree, it registers the utility values of the corresponding node and this node’s ancestors in the transaction. If a node has already registered with a utility value, the algorithm just adds the new value to it. Sub-trees are constructed by the same way, i.e., each node does not contain utility values of its children nodes. So UP-Growth’s over-estimated utility value (used for judging whether an itemset is a candidate) is lower than that of IHUP. UP-Growth effectively reduces the number of candidates and improves the time efficiency of identifying of candidates.

### 2.2 One phase algorithms

The main shortcoming of Two-phase algorithms is that they generate a large number of candidates including non-existing itemsets of the dataset, and they need multiple scans on the original dataset. To address this main shortcoming, some one-phase algorithms have been proposed.

HUI-Miner [15] firstly introduced the *utility-list* structure. Then some algorithms based on the *utility-list* structure have been proposed, such as FHM [22], HUP-Miner [23], mHUIMiner [24], and ULB-Miner [9]. Algorithm FHM [22] applied a depth-first search to find high utility itemsets, and was shown to be up to seven times faster than HUI-Miner. Algorithm mHUIMiner [24] combined ideas from the HUI-Miner and IHUP algorithms to efficiently mine high utility itemsets from sparse datasets. Algorithm ULB-Miner [9] extended algorithm FHM [22] and HUI-Miner [15] by utilizing a utility list buffer structure, which helped reduce the memory and runtime usage of FHM algorithm.

Algorithm D2HUP [8] directly found high utility itemsets without generating candidates based on pattern-growth, represented the databases using a hyper structure, and was shown to be up to one order of magnitude faster than UP-Growth.

Algorithm EFIM [7] directly found high utility itemsets without generating candidates. It applied a horizontal database representation for storing itemset information to reduce memory usage, and utilized the concepts of transaction merging, database projection, and fast utility computation for mining high utility itemsets. The experimental results showed that algorithm EFIM was 2 to 3 order of magnitudes faster than these algorithms HUI-Miner [15], UP-Growth [10], FHM [22], and HUP-Miner [23].

IMHUP [25] uses an indexed utility list for mining HUIs, and neither stores transaction identifiers nor perform costly transaction list intersections. IMHUP algorithm is about 2-12 times faster than FHM algorithm. But IMHUP algorithm is not superior to EFIM algorithm [6]. HMiner [6] is based on algorithm HUI-Miner [15], adopts a compact utility list for merging transactions containing all extended items, avoids the expensive cost of computing, and finally gets obviously performance improvement.

### 2.3 Variant algorithms

Based on existing research on HUI mining, several variant algorithms have been proposed, e.g., high average-utility mining [26–28], Top-K high utility mining [29, 30], HUI mining from data stream [31, 32], high-utility association rules [33], multi-core or parallel mining [34, 35], and HUIM over multiple data sources [36]. Most of these studies mainly apply methods of one phase or two phase.

### 2.4 Differences from previous works

The pattern-tree based algorithms [10, 16, 19] mentioned above discard utility value of individual item of a transaction. They cannot retrieve the exact utility value of an itemset, and must utilize an over-estimated utility value to generate candidate itemsets. It is obvious that the smaller the over-estimated utility threshold is, the lesser the candidates will be, and the better the performance of the mining algorithm may achieve. If we can get the exact utility value of an itemset, we can identify directly whether it is a HUI without bothering the processing of candidates. For this reason, we construct a novel tree structure for mapping transaction itemsets, the itemsets’ exact utility values can be retrieved from the tree. In summary, our study adopts this pattern-growth approach to mine HUIs from a tree without generating candidate itemsets.

## 3 Preliminaries

In this section, we give the definition of the HUI mining.

### 3.1 Basic concepts

Given a set of *m* unique items *I* = {*i*_1_, *i*_2_, …, *i*_*m*_}, an itemset *X* ⊆ *I* containing *k* distinct items is called a *k*-itemset. A transaction dataset *DB* = {*T*_1_, *T*_2_, …, *T*_*n*_} contains *n* transactions. Each transaction *T*_*d*_ (*d* = 1, 2, …, *n*) involves a subset of all unique items in *I*, called a transaction itemset. For convenience, we use the notation *T*_*d*_ represent the transaction itemset.

For a utility-valued transaction database, each item *i*_*r*_ (*r* = 1, 2, …, *m*) has a unit profit $p(i_{r})\in R$, and each item *i*_*r*_ in a transaction *T*_*d*_ is attached with a quantity $q(i_{r},T_{d})\in R$ with its occurrence in the transaction (e.g., quantity purchased, dollar amount paid, or profit from the transaction).

**Definition 1** (Item Utility). *The utility of the item* *i*_*r*_ *in a transaction* *T*_*d*_ *is denoted as* *u*(*i*_*r*_, *T*_*d*_) *and calculated as*

$$\begin{array}{c} u\left(i_{r},T_{d}\right)=p\left(i_{r}\right)*q\left(i_{r},T_{d}\right), \end{array}$$ (1)

*where p*(*i*_*r*_) *is the unit profit of item i*_*r*_, *and* *q*(*i*_*r*_, *T*_*d*_) *is the quantity of item i*_*r*_’s *occurrence in transaction T*_*d*_, ∀*i* = 1, 2, …, *m*, ∀*d* = 1, 2, …, *n*.

**Definition 2** (Itemset Utility). *The utility of an itemset X in a transaction T*_*d*_ *is denoted as u*(*X*, *T*_*d*_) *and calculated as*

$$\begin{array}{c} u\left(X,T_{d}\right)=\{\begin{array}{cc} 0, & if\;X⊈T_{d};\\ \sum_{i_{r}\in X}u\left(i_{r},T_{d}\right), & if\;X\subseteq T_{d}; \end{array} \end{array}$$ (2)

*where u*(*i*_*r*_, *T*_*d*_) *is the utility of the item i*_*r*_ *in transaction* *T*_*d*_. *The utility of the itemset X in the whole transaction dataset DB* = {*T*_1_, *T*_2_, …, *T*_*n*_} *is denoted as u*(*X*) *and calculated by*

$$\begin{array}{c} u\left(X\right)=\sum_{T_{d}\in DB}u\left(X,T_{d}\right). \end{array}$$ (3)

Since a transaction corresponds to a transaction itemset, the transaction utility is a special case of itemset utility. More specifically, the utility of a transaction *T*_*d*_ is denoted as *tu*(*T*_*d*_) and calculated by

$$\begin{array}{c} tu\left(T_{d}\right)=\sum_{i_{r}\in T_{d}}u\left(i_{r},T_{d}\right). \end{array}$$ (4)

**Definition 3** (Support Number). *The support number (sn) of an itemset X is the number of transaction itemsets containing X*.

**Definition 4** (Transaction-Weighted Utility). *The transaction-weighted utility of an itemset X is denoted as TWU(X), and is calculated by*

$$\begin{array}{c} TWU\left(X\right)=\sum_{T_{d}\in \left\{T\in DB:X\subseteq T\right\}}tu\left(T_{d}\right). \end{array}$$ (5)

*TWU*(*X*) is the sum of the transaction utilities of all transaction itemsets containing *X*.

**Example 1** (Utility-Valued Transaction Database). *The first two columns in* Table 1 *and the first two columns in* Table 2 *provide an example utility-valued transaction database*. *More specifically*, Table 1 *is a dataset containing 7 transaction itemsets, and* Table 2 *shows the unit profit value of each item in* Table 1.

**Table 1:** An example database.

TID	Items and Quantities	*tu*(*T*_*i*_)	*MU*(∅, *T*_*i*_, 3)	*u*(*B*, *T*_*i*_)	*u*(*C*, *T*_*i*_)	*u*({*B*, *C*}, *T*_*i*_)
*T*_1_	(*B*, 4)(*C*, 3)(*D*, 3)(*E*, 1)	24	21	12	3	15
*T*_2_	(*B*, 2)(*C*, 2)(*E*, 1)(*G*, 4)	15	13	6	2	8
*T*_3_	(*B*, 3)(*C*, 4)	13	13	9	4	13
*T*_4_	(*A*, 1)(*C*, 1)(*D*, 2)	15	15	0	1	0
*T*_5_	(*A*, 2)(*B*, 2)(*C*, 2)(*D*, 2)(*E*, 1)(*F*, 9)	44	35	6	2	8
*T*_6_	(*A*, 1)(*C*, 6)(*D*, 2)(*E*, 1)(*G*, 8)	31	24	0	6	0
*T*_7_	(*A*, 2)(*C*, 4)(*D*, 3)	30	30	0	4	0

**Table 2:** Profit table.

Item	Profit	*MU*	*sn*
*A*	10	104	4
*B*	3	82	4
*C*	1	151	7
*D*	2	125	5
*E*	3	93	4
*F*	1	35	1
*G*	1	37	2

**Definition 5** (Promising Itemset). *An itemset/item X is called a promising itemset/item for high utility itemsets/item if* *TWU*(*X*) ≥ *min*_*uti* (*min*_*uti* *is a user-specified minimum utility value)*, *otherwise it is an unpromising itemset/item*. *A promising itemset is also called a candidate itemset for HUIs*.

**Lemma 1** (Transaction-Weighted Downward Closure Property). *Any subset of a promising itemset is a promising itemset and any superset of an unpromising itemset is an unpromising itemset*.

Lemma 1 has been proved in [13]. For example, if {*ACD*} is a promising itemset, the itemset {*AC*} (or any sub itemset of {*ACD*}) is also a promising itemset. On the other hand, if {*AC*} is unpromising, all its super itemsets (such as {*ACD*}) are unpromising.

**Theorem 1**. *Let item Q be an unpromising item in dataset* *DB*, *then any itemset X containing Q is not a high utility itemset* [10].

*Proof*. According to Lemma 1, itemset *X* is an unpromising itemset. According to Definition 2 and 4, *u*(*X*) ≤ *TWU*(*X*), the utility of itemset *X* is less than the minimum utility value, thus itemset *X* is not a HUI.

**Definition 6** (Maximum Utility of transaction). *The transaction utility of an itemset X with* *k*-*length is denoted as* *mu*(*X*, *T*_*d*_, *k*), *and is calculated by*

$$\begin{array}{c} mu\left(X,T_{d},k\right)=u\left(X,T_{d}\right)+\max\{\sum_{i=1}^{\min(k,|T_{d}|)}u(x_{i},T_{d})|x_{i}\in T_{d}\wedge x_{i}\notin X\} \end{array}$$ (6)

**Definition 7** (Maximum Utility). *The maximum utility of an itemset X with* *k*-*length is denoted as* *mu*(*X*, *k*), *and is calculated by*

$$\begin{array}{c} mu\left(X,k\right)=\sum_{X\in T_{d}\wedge T_{d}\in DB}mu\left(X,T_{d},k\right). \end{array}$$ (7)

**Theorem 2**. *Let mu*(*X*, *k*) *be less than the minimum utility threshold in dataset* *DB*, *then any l*-*itemset Y*(*l* ≤ *k*) *containing X is not a high utility itemset*.

*Proof*. According to Eqs 4 and 6, *mu*(*X*, *T*_*d*_, *k*) ≤ *tu*(*T*_*d*_) and *tu*(*Y*, *T*_*d*_) ≤ *mu*(*X*, *T*_*d*_, *k*). So for any itemset *X* in dataset *DB*, *mu*(*X*, *k*) ≤ *TWU*(*X*) and *tu*(*Y*) ≤ *mu*(*X*, *k*).

**Definition 8** (Remain Transaction-itemset). *Given a transaction itemset* *T*_*d*_ = {*x*_1_, *x*_2_, ⋯, *x*_*i*_, ⋯}, *and an ordered subset* *X of itemset T*_*d*_ $\left(X=\{x_{i},x_{i_{1}},x_{i_{2}},\cdots ,x_{i_{j}}\}\right)$, then itemset {*x*_1_, *x*_2_, ⋯, *x*_*i*−1_} *is named remain transaction-itemset of X in T*_*d*_, *and denoted as rt*(*X*, *T*_*d*_).

**Definition 9** (Remain Transaction-Weighted Utility). *The remain transaction-weighted utility of an itemset X is denoted as RTWU(X), and is calculated by*

$$\begin{array}{c} RTWU\left(X\right)=\sum_{T_{d}\in \left\{T\in DB:X\subseteq T\right\}}tu\left(rt\left(X,T_{d}\right)\right). \end{array}$$ (8)

**Definition 10** (Remain Maximum Utility). *The remain maximum utility X with k*-*length is denoted as RMU*(*X*, *k*), *and is calculated by*

$$\begin{array}{c} RMU\left(X,k\right)=\sum_{X\in T_{d}\wedge T_{d}\in DB}mu\left(X,rt\left(X,T_{d}\right),k\right). \end{array}$$ (9)

### 3.2 Problem definition

In a transaction dataset, an itemset is a *high utility itemset* if its utility value is not less than a user-specified minimum utility value, where the utility of an item in a transaction is defined as its internal utility multiplied by its external utility. The utility of an itemset in a transaction is defined as the sum of its all items’ utility in the transaction. For example, the utility of an itemset *X* in a transaction dataset is defined as the sum of its utility in each transaction containing *X*.

**Definition 11** (High Utility Itemset). *An itemset X is called a high utility itemset if its utility* (*u*(*X*)) *is not less than a user-specified minimum utility value*.

Given a transaction database *DB*, the problem of HUI mining aims at finding all HUIs from *DB*. Mining HUIs from a database also refers to finding all itemsets whose utility value is not less than a user-specified minimum utility value.

## 4 Proposed algorithms

Several algorithms have been proposed to mine HUIs based on pattern-growth, but they can not mine HUIs without generating candidate itemsets. This paper proposed an algorithm HUIL-TN (High-Utility Itemsets mining based on Length and Tail-Node tree) for mining HUIs by using pattern-growth without generating candidates.

**Algorithm 1**: Algorithm HUIL-TN

 **Input**: *DB*: transactions data; *η*: minimum utility threshold; *k*: the maximum length of high utility itemsets in *DB*.

 **Output**: HUIs

1 find the maximum length *k* of HUIs on part of *DB*;

 // create global TN-tree *T* and Header Table *H*

2 CreateGTree(*DB*, *η*, *k*);

 // find all HUIs, which lenth is not more than *k*, from the TN-tree *T*

3 MHUIs(*T*, *H*, base-itemset, *k*);

Algorithm HUIL-TN is shown in Algorithm 1, and includes three parts:

- First, we randomly select some data to find the maximum length *k* of HUIs (Line 1), and use *k* to estimate the maximum length of HUIs in *DB*.
- Second, we map the dataset *DB* to a global TN-tree and header table (Line 2).
- Finally, we mine HUIs from the global tree (Line 3). We explain in detail the process of creating a global tree and mining HUIs from the global tree in the following subsections.

Section 4.1 describes constructing process of tree using transaction datasets, including description of tree structure(4.1.1) and tree construction(4.1.2). Section 4.2 describes algorithm of mining HUIs from a tree. Section 4.3 describes comparison with existing Algorithms. Section 4.4 gives algorithm analysis.

### 4.1 Constructing trees for maintaining data

To facilitate the mining process and avoid scanning the dataset many times, a tree structure is employed to maintain the dataset in our algorithm. In this subsection, we firstly introduce a new tree structure called TN-tree (Tail-Node tree) to maintain a transaction dataset, and then we describe the algorithm of mining HUIs from the TN-tree.

#### 4.1.1 The structure of TN-tree

In this study, we propose a new data structure TN-tree for storing critical utility information from the dataset for HUI mining. TN-tree can be used to store the utility values of itemsets. Utility value of an itemset can be retrieved from the TN-tree and can be used to determine whether this itemset is a HUI.

Like other tree-structures for pattern generation, in a TN-tree, each node *N* contains the following fields:

- *N*.*name*: item name of the node *N*,
- *N*.*parent*: parent node of the node *N*, and
- *N*.*children*: a set of the children nodes of *N*.

**Definition 12** (Tail-node). *When a transaction is inserted to a tree, its last node is called a Tail-node of this transaction*.

**Definition 13** (Path-itemset). *A set of items on path that is from a node to root is called path-itemset of this node*.

In order to get utility value of each itemset from the tree, a tail-node contains the following fields in addition:

- *N*.*piu* is a list which records each item utility in a path-itemset;
- *N*.*bu* is the utility of the base-itemset in a path-itemset.

*N*.*bu* and *N*.*piu* are called the *tail-information* of node *N*. The tail-information is important, because all itemsets that potentially have a utility score above the minimum utility threshold can be found by using tail-information stored on the tree.

**Fig 1:**
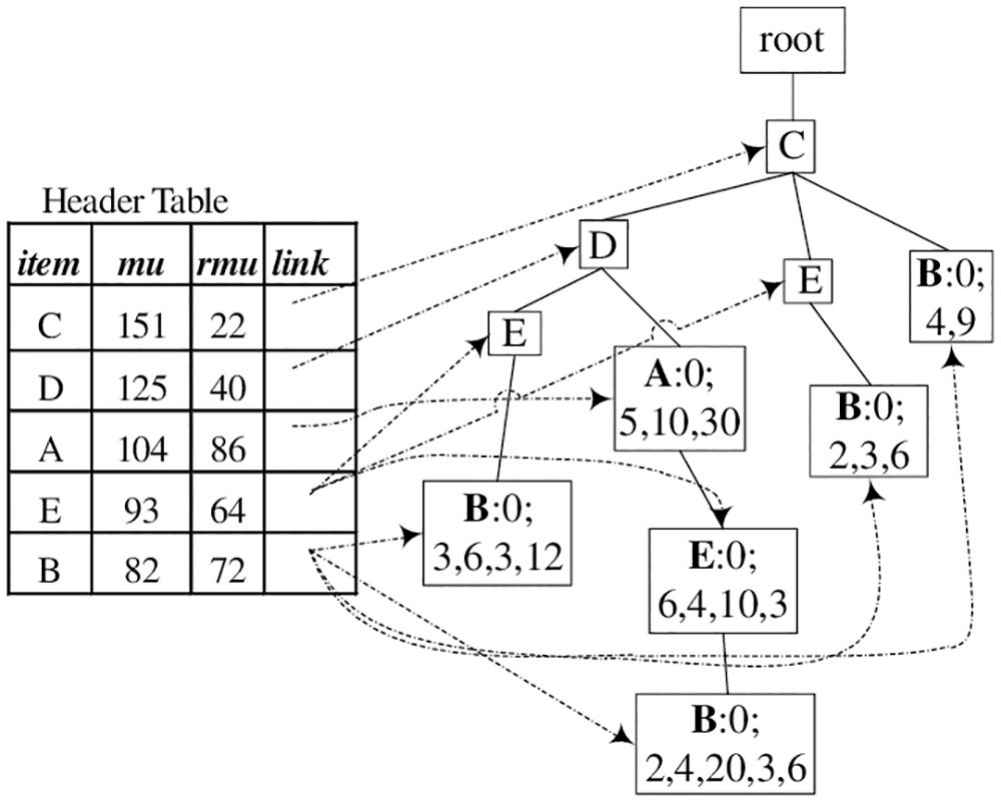
Example TN-tree constructed using data in Tables 1 and 2.

Fig 1 illustrates an example TN-tree, which was constructed based on data in Tables 1 and 2. For example, the leftmost node *B* is a tail-node of the itemset {*C*, *D*, *E*, *B*}, and the sequence of numbers, “3, 6, 3, 12” on the node, represent the total utility values of items *C*, *D*, *E*, and *B* in the entire dataset, respectively.

#### 4.1.2 TN-tree construction

A TN-tree can be constructed by two scans of a transaction dataset. The pseudocode is provided in Algorithm 2.

**Algorithm 2**: CreateGTree

 **Input**: *DB*: transactions data; *η*: minimum utility threshold; *k*: the maximum length of high utility itemsets in *DB*.

 **Output**: the TN tree *T*

 // First scan of the database DB

1 Initiate a header table *H* containing the fields of item ID, *TWU*, *sn*, and links;

2 **for** *each transaction TS of DB* **do**

3  **for** *each item I in TS* **do**

4   *H*.*I*.*mu*+ = *mu*(∅, *TS*, *k*);

5  **end**

6 **end**

7 Delete unpromising items from *H* based on threshold *η*;

8 Sort *H* by the descending order of *mu* of *H*;

 // Second scan of the database *DB*

9 Initialize a TN-tree *T* with an empty root node;

10 **for** *each transaction TS of DB* **do**

11  Delete unpromising items from *TS*;

12  Sort items of *TS* according to *H*, with utility values, to *X*;

13  Insert *X* to *T*;

   // Process the tail-information for the tail-node

14  *N*.*piu*+ = *X*.*piu*; // element-by-element addition

15  *H*.*N*.*RMU*+ = *RMU*(*N*, *X*, *k*);

16 **end**

In the first scan of dataset, we create a header table. We first compute the *mu* value of each unique item in the dataset. The items of the header table are then arranged in the descending order of *mu* values (or *TWU* values, or support number). Unpromising items are then deleted from the header table.

In the second scan, transaction itemsets are added into the TN-tree. The TN-tree is initialized as an empty root node (i.e., its parent node and item name are null). For each transaction in the dataset, we take the following process:

1. Delete unpromising items from the transaction itemset (Line 11).
2. Sort the remaining promising items according to their ordering in the header table and create a sorted itemset *X* (Line 12).
3. Add itemset *X* into the TN-tree, and store the number of itemset *X*, and the utility of each item in *X* to the tail-node of *X*, and store the *RMU* values of all items in *X* and link of new nodes to the header table.

Note that the field *bu* on each tail-node is initialized as 0 in this (global) TN-tree. Its value will be updated in the HUI mining process when sub-trees are constructed (see the following subsection).

Example 2 illustrates the construction process of a TN-tree using the dataset in Tables 1 and 2.

**Example 2** (TN-tree Construction). *Suppose the minimum utility value* *min*_*uti* *is* 70.

**Fig 2:**
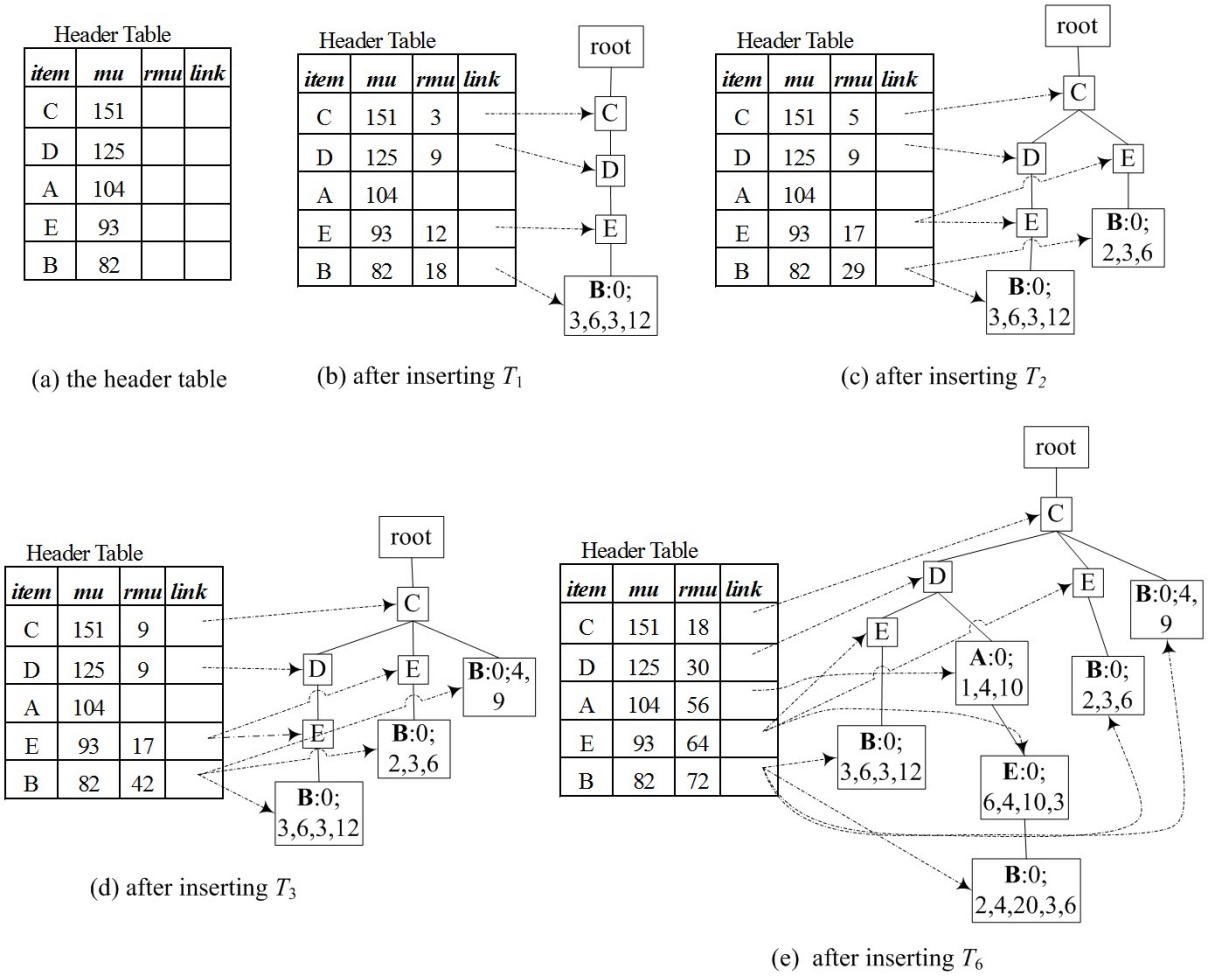
Construction of a TN-tree.

*Firstly, a header table H is created by one scan of the dataset. The result is shown in* Fig 2(a).

*Then, a TN-tree is initialized as a root node whose parent node and item name are null. A second scan of the dataset will add all transactions to the TN-tree by the following process*.

1. *For the first transaction itemset* {*B*, *C*, *D*, *E*}, *we remove unpromising items from the itemset and sort items of the itemset according to the order of H*. *Then we get the itemset* {*C*, *D*, *E*, *B*}, *add the itemset to a TN-tree, and store the piu values* (3, 6, 3, 12) *to the field piu on the tail-node*. *The TN-tree is shown in* Fig 2(b) *after T*_1_ *is added to the tree, where node B is a tail-node, and* 0; 3, 6, 3, 12 *shows its bu* = 0, *and piu* = {3, 6, 3, 12}. *We also modify RMU values of each item of the itemset* {*C*, *D*, *E*, *B*}, *as shown the header table in* Fig 2(b).
2. *For the second transaction itemset* {*B*, *C*, *E*, *G*}, *we remove unpromising item G*, *sort items of the itemset according to the order of H*, *and get the itemset* {*C*, *E*, *B*}. Fig 2(c) *shows the TN-tree and the header table after T*_2_ *was added to the tree*.
3. *For the third transaction itemset* {*B*, *C*}, *we obtained the sorted itemset* {*C*, *B*}. Fig 2(d) *shows the TN-tree and the header table after T*_3_ *was added to the tree*.
4. *By the above method, the first six transactions were added to the TN-tree*. *The result was shown in* Fig 2(e).
5. *After all the transaction itemsets were added to the tree, the TN-tree was shown as* Fig 1. *When T*_7_ *was added to the tree, there was a path “root*-*C*-*D*-*A” on the TN-tree and node A was also the tail-node in* Fig 2(e). *Therefore, we just need to modify the tail-information on the tail-node A, and modify RMU values of each item of the itemset*. *The modified tail-information and the header table is shown in* Fig 1.

### 4.2 Mining HUIs from a TN-tree

In this section, we firstly introduce the concept of sub-tree, then describe and analyze the proposed algorithm.

Like algorithm FP-growth [2], algorithm HUIL-TN applies a recursive method that iterates over sub-tree of the global TN-tree initially constructed. To clarify the description of HUI-TN, we firstly give the following definitions.

**Definition 14** (Base-Itemset and Conditional Tree). *A* conditional tree *(also called a sub-tree)* [2] *of itemset X is a tree that is constructed using all transaction itemsets containing itemset X* (*X is removed from these transactions itemsets before they are added to the conditional tree*). *Itemset X is called the* base-itemset *of this conditional tree*.

A tree that is constructed by all transaction itemsets of a dataset and, whose base-itemset is null, is called a global tree. A global tree is also called a conditional tree whose base-itemset is null. *u*(*X*, *t*) in a transaction itemset *t* containing *X* is also called base-utility (abbreviated as *bu*) of transaction itemset *t* in the conditional tree *T*.

**Definition 15** (Sub Dataset). *In a conditional tree T whose base-itemset is X* (*if X is null, T is a global tree*), *suppose item Q appears in k tail-nodes*, *and the corresponding path itemsets are Y*_1_, *Y*_2_, …, *Y*_*k*_, *the itemsets* *Y*_1_ ∪ *X*, *Y*_2_ ∪ *X*, …, *Y*_*k*_ ∪ *X* (*along with their utility values*) *constitute the* sub dataset *of itemset* {*Q*} ∪ *X*. *Each record in sub dataset is called* sub transaction-itemset.

**Definition 16** (Local Candidate). *If the* MU *value of an item in a sub dataset is less than the minimum utility value*, *it is called a* local unpromising item (*local non-Candidate*); *otherwise, it is called a* local promising item (*local candidate*).

According to Theorem 2, algorithm HUIL-TN removes all unpromising items from original transaction itemsets when it creates the TN-tree with transaction itemsets, and removes all local unpromising items of a sub dataset when it creates a sub TN-tree.

The algorithm of mining HUIs from a TN-tree is shown in Algorithm 3.

We process each item (denoted as *Q*) in the header table *H*, starting from the last item, by the following steps.

First, if *RMU* is less than the predefined minimum utility value, go to the next step; otherwise, we add item *Q* to a base-itemset (which is initialized as ∅) and generate HUI and create sub TN-tree to perform mining recursively (Lines 15-16). More specifically, if (*BU*+ *NU*) is not less than the predefined minimum utility value, then the current base-itemset is a HUI (Lines 9-11); if there is only one node for the item *Q*, we do not generate sub TN-tree and directly process the path-itemset of this node(line 13). We remove the item *Q* from the current base-itemset after we perform a recursive mining process on the new sub TN-tree (line 18).

Then, for each of these *m* tail-nodes (which we denote as *N*_*i*_, *i* = 1, 2, …, *m*), we modify its tail-information by deleting item Q’s utility from list *N*_*i*_.*piu*. If its parent node contains a tail-information, then accumulate this tail-information to its parent’s tail-information (lines 26-27); otherwise move this tail-information to its parent(lines 23-24).

The propose of Algorithm 4 is to find all HUIs from subsets of itemset *X* when itemset *X* is a HUI. First, generate a HUI *X* (line 1). We remove one item from X in turn and obtain an new itemset *Y* (lines 5-6). We will recursively process itemset *Y* (line 7) if the utility value of itemset *Y* is less than the minimum utility value *minutil*.

The constructing process of sub tree is summarized in Algorithm 5, is as follows. First, we create a new header table *subH* by scanning the corresponding path-itemsets in the current TN-tree (line 3), including deleting unpromising items from *subH* and sorting its items in the descending order of *RMU* (lines 6-7). Second, we process each path-itemsets in the current TN-tree, including deleting unpromising items(line 13), sorting items according to *subH* (line 14), and inserting the path-itemsets to a new TN-tree *subT* (lines 15-17).

**Algorithm 3**: MHUIs

**Input**: *T*: the TN-tree constructed from the transactions data; *H*: the header table constructed from the transactions data; base-itemset; *k*: the maximum length of HUIs.

**Output**: HUIs

1 **foreach** *item Q in H (with a bottom-up sequence)* **do**

  // Step 1: Generate HUIs and create sub TN-tree

2  **if** *RMU* ≥ *min*_*util* **then**

3   base-itemset = base-itemset ∪{*Q*}

   // Calculate BU and NU

4   *BU* = 0, *NU* = 0;

5   **foreach** *node N of item Q in T* **do**

6    *BU* = *BU* + *N*.*bu*; // base itemset utility

7    *NU* = *NU* + *N*.*nu*; //*N*.*nu* is the utility of item *Q* in the list *N*.*piu*

8   **end**

9   **if** *BU*+ *NU* ≥ *min*_*util* **then**

10    Copy base-itemset to HUIs; // generate one HUI

11   **end**

12   **if** *there is one node N for the item Q* **then**

13    CreateHuibyOneTr(path-itemset of *N*, *N*.*piu*, 0);

14   **else**

15    Create a sub TN-tree *subT* and a header table *subH* for base-itemset (see Algorithm 5);

16    MHUIs(*subT*; *subH*, base-itemset, *k*-1); // recursive call

17   **end**

18   Remove item *Q* from itemset base-itemset;

19  **end**

   // Step 2: Remove item *Q* from tree *T* by moving each tail-node’s tail-information to its parent

20  **foreach** *node N of item Q in T* **do**

21   Remove utility value of item *Q* from list *N*.*piu*;

22   **if** *N*.*parent*.*bu*==*NULL* **then**

23    *N*.*parent*.*bu* = *N*.*bu*;

24    *N*.*parent*.*piu* = *N*.*piu*;

25   **else**

26    *N*.*parent*.*bu* = *N*.*parent*.*bu* + *N*.*bu*;

27    *N*.*parent*.*piu* = *N*.*parent*.*piu* + *N*.*piu*;

28   **end**

29   Remove node *N* from *T*;

30  **end**

31 **end**

32 **return** *HUIs*;

**Algorithm 4**: Procedure CreateHuibyOneTr

**Input**: *X*: an itemset; *U*_*X*: utility value of each item in *X*; *p*: index of *X*.

**Output**: *HUIs*

1 *X* is a HUI;

2 *sumUtility* = sum of utility values of all items in *X*;

3 **for** *int* *i* = *p*; *i* < *X*.*length*; *i++* **do**

4  if *sumUtility*—*U*_*X*[*i*] < *minutil* then continue;

5  copy *X* into *Y* excluding item *x*;

6  copy *U*_*X* into *U*_*Y* excluding the utility of item *x*;

7  CreateHuibyOneTr(*Y*, *U*_*Y*, *i*);

**end**

**Example 3** (HUI Mining based on TN-tree). *For example, in* Fig 1, *item “B” is the last item in the header table*. *Because the RMU value 72 is not less than the minimum utility value 70, we firstly add item “B” to a base-itemset (initialized as null), resulting base-itemset B, and calculate its BU* = 0, *NU* = 12 + 6 + 6 + 9 = 33. *Because BU* + *NU* = 33 < 70, *this itemset* {*B*} *is not a high utility itemset*. *Then we still construct a sub header table and a sub TN-tree for the current base-itemset* {*B*}.

**Fig 3:**
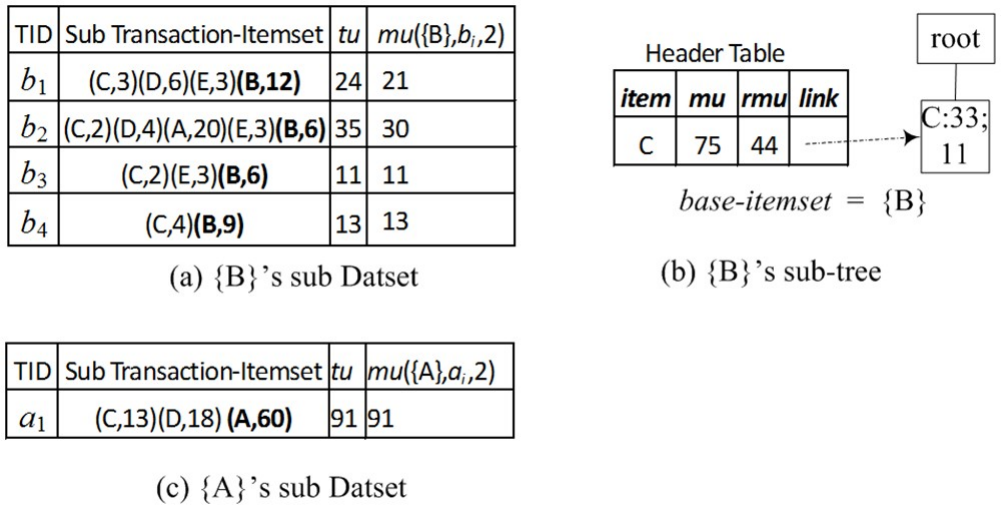
The HUIL-TN algorithm for mining high utility itemsets.

*A sub header table is created as the following*. *From the path “root*-*C*-*D*-*E*-*B*: 1, 0; 3, 6, 6, 12; 24” *in* Fig 1, *get an itemset* {*C*, *D*, *E*, *B*}, *and utility of items C*, *D*, *E*, *B* (i.e., 3, 6, 3, 12), *respectively*. *See the first sub transaction-itemset of the sub dataset in* Fig 3(a). *Similarly, we can get other three sub transaction-itemset from the other three paths, respectively: root*-*C*-*D*-*A*-*E*-*B*: 1, 0; 2, 4, 20, 3, 6, *root*-*C*-*E*-*B*: 1, 0; 2, 3, 6, *and root*-*C*-*B*: 1, 0; 4, 9. *See the sub dataset in* Fig 3(a) (*the number associated with each item, such as 3 in* (*C*, 3), *is the utility value of this item*).

*A sub header table is created by scanning the sub dataset in* Fig 3(a), *the result is shown in* Fig 3(b). *A sub header table just maintains all local promising items*. *A sub TN-tree is created by the method of TN-tree in subsubsection 4.1.2, except that the utility values of itemset* {*B*} *of each sub transaction-itemset in* Fig 3(a) *are accumulated to the field bu on the tail-node and the item B is not added to the sub TN-tree*. *The result is shown in* Fig 3(b).

*Then we perform a recursive mining process on the new sub header table and sub TN-tree*. *For the last item C in the header table* (see Fig 3(b)): *because RMU* < 70, *item C is not added to the base itemset, and no new sub TN-tree or HUI is generated*.

*After processing all items in* Fig 3(b), *we go on processing remaining items of the header table in* Fig 1. Fig 3(c) *is the sub dataset of itemset* {*A*}. *Because there is one transactoin in* Fig 3(c), *no sub header or no sub TN-tree is created; i.e*., {{*AD*}, {*ACD*}, {*AC*}}.

**Fig 4:**
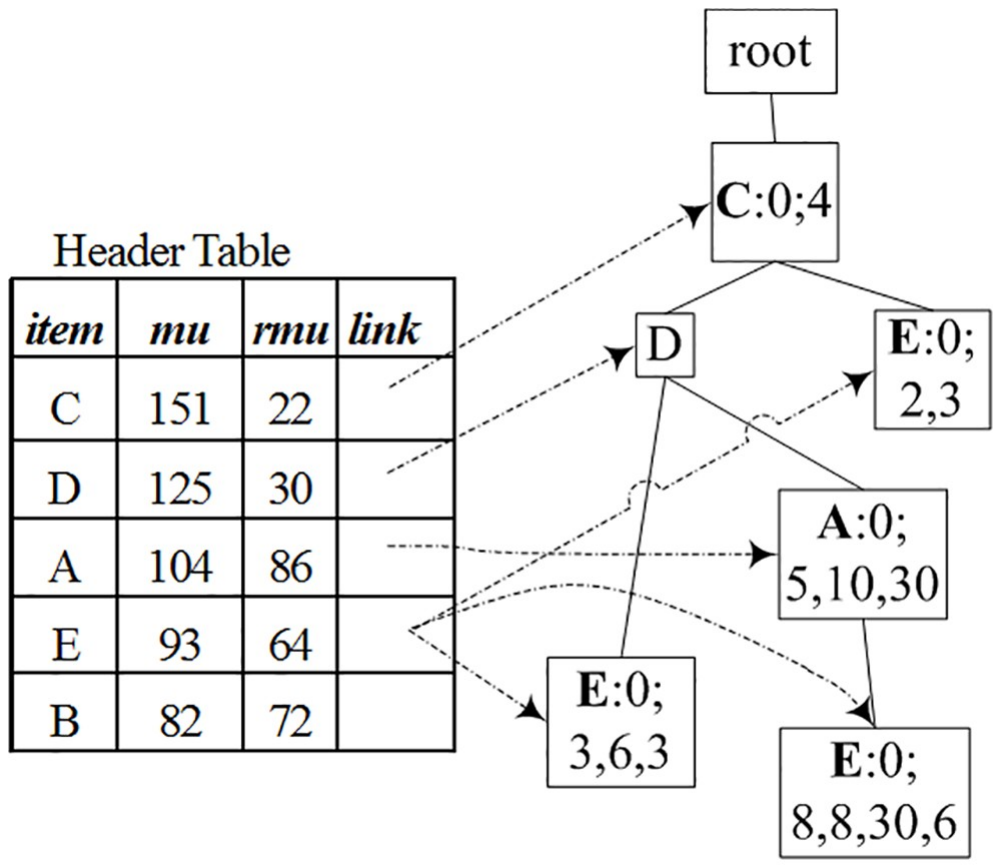
After removing node B.

The “add/move” process (Step 2 of Algorithm 3) is a key operation of this algorithm. When a transaction itemset (or sub transaction-itemset) is added to a TN-tree, its base-utility and each item’s utility are stored in its tail-node, not in the node itself. Moreover, since a node can appear in multiple branches, its base-utility, utility, etc., should be the sum of the corresponding values of all its tail-nodes. So tail-information of one node should be passed to its parent node after this node is processed. For example, after processing node *B*: 0; 3, 6, 3, 12 in Fig 1, according to Step 3, remove *B*’s utility (12) from *B*.*piu* (3, 6, 3, 12), resulting a new tail-information 0; 3, 6, 3. Since *B*’s parent node *E* does not contain tail-information, we move this new tail-information to this node *E*, resulting in *E*: 0; 3, 6, 3 (see Fig 4). In the same manner, tail-nodes *B*: 0; 2, 3, 6 and *B*: 0; 4, 9 in Fig 1 were processed and moved to their parent nodes, resulting in *E*: 0; 2, 3 and *C*: 0; 4. Tail-node *B*: 0; 2, 4, 20, 3, 6 was added to its parent node (because its parent node contains tail-information), resulting in *E*: 0; 8, 8, 30, 6, see Fig 4.

**Algorithm 5**: Create sub header table and sub TN-tree

 **Input**: the previous TN-tree *T*, item *Q*, the length *k*

 **Output**: *subT* and *subH*

 // Construct *subH*

1 **foreach** *node N of item Q in T* **do**

2  **foreach** *item I in path-itemset N in T (excluding Q)* **do**

3   *H*.*I*.*MU* = *H*.*I*.*MU*+ *N*.*MU*;

4  **end**

5 **end**

6 Delete unpromising items from *subH*;

7 Sort *subH* in the descending order of *MU* of *subH*;

 // Construct *subT*

8 Initialize *subT* with an empty root node;

9 **foreach** *node N of item Q in T* **do**

10  *X* = path-itemset of *N* (excluding Q);

11  *u* = the utility of item *Q* in *N*.*piu*;

12  *Tin* = tail-information of *N*;

13  Delete unpromising items that is not in *subH* from *X* and modify *Tin*;

14  Sort items of *X* according to *subH* and modify *Tin*;

15  Insert *X* to *subT*;

  // Process the tail-information of the tail-node *sN* of itemset *X*

16  *sN*.*piu* = *sN*.*piu* + *Tin*.*piu*;

17  *sN*.*bu* = *sN*.*bu* + *Tin*.*bu*+ *u*;

18 **end**

The above algorithm HUIL-TN creates global header table or sub-header table by using *MU* or *RMU* values. Moreover, we also design algorithm HUI-TN that creates global header table or sub-header table by using *TWU* or *RTWU* values.

### 4.3 Comparison with algorithms based on pattern-tree

**Fig 5:**
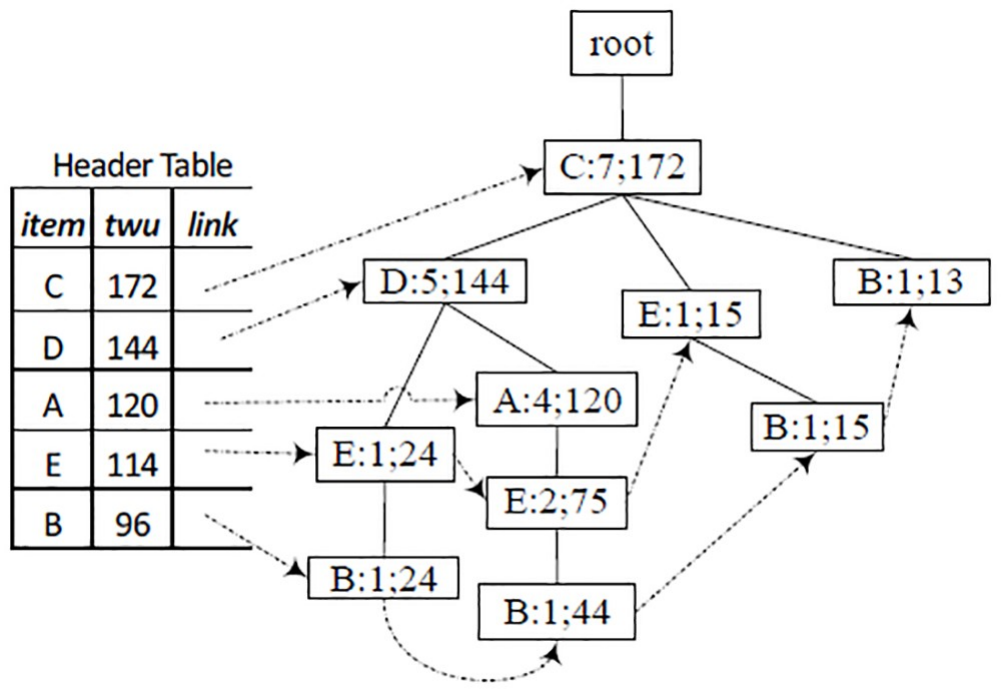
IHUP-Tree [16] based on toy data in Tables 1 and 2 (*η* = 70).

**Fig 6:**
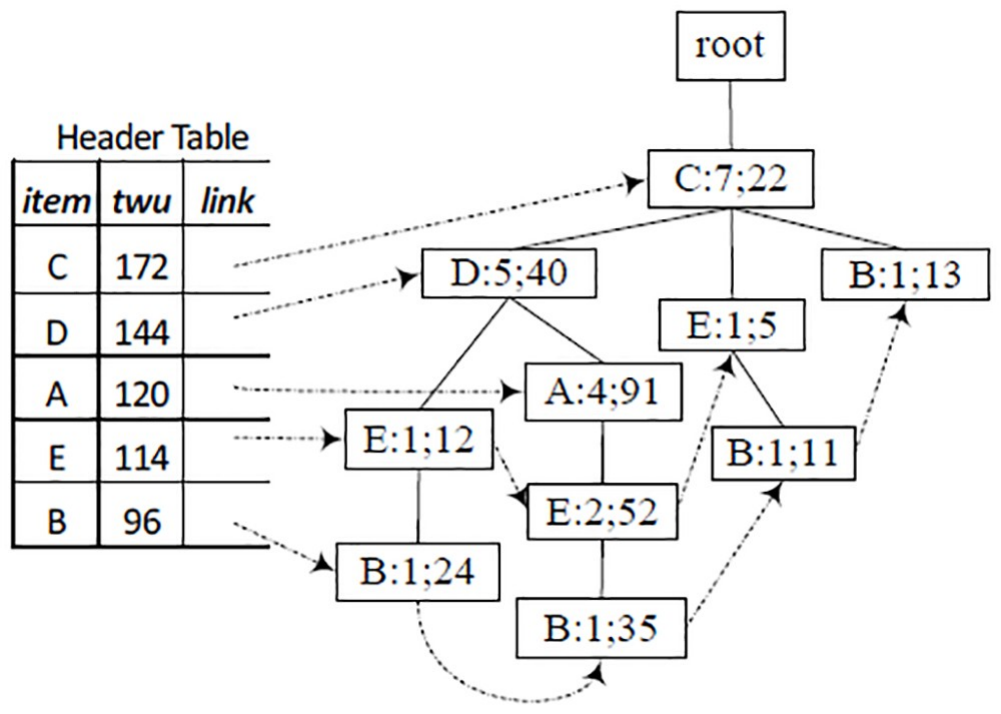
UP-Tree [10] based on toy data in Tables 1 and 2 (*η* = 70).

Tree structures have been used to represent transaction databases for pattern mining. For example, for the dataset in Table 1 and the profit table in Table 2, a global IHUP-Tree is shown in Fig 5, in which items are arranged in the descending order of *TWU* values. In the second step, IHUP generates candidates for HUIs from the IHUP-Tree by employing the FP-Growth method [2]. In the third step, IHUP scans the dataset to find all HUIs from the candidates. During the construction of a UP-Tree Fig 6, the unpromising items and their utilities are eliminated from the transaction utilities, and the utilities of its descendants of any node are discarded from the utility of the node. For any itemset *X*, the value of TWU(*X*) in the UP-Tree is not bigger than that in the IHUP-Tree, so the number of candidates created by the algorithm UP-Growth is not bigger than that created by the algorithm IHUP.

The structures of the header table in algorithms IHUP and UP-Growth contains item, *TWU* value and link information, as shown in Figs 5 and 6. The structures of IHUP-Tree and UP-Tree are identical: each node on them contains item, support number, *TWU* (or a value derived from *TWU* value), link to parent, link to children, and link to the next node.

When a transaction itemset is inserted to a UP-tree, each node does not contain utility values of its children nodes. So UP-Growth’s over-estimated utility value (used for judging whether an itemset is a candidate) is lower than that of IHUP. So this effectively reduces the number of candidate and improves the time efficiency of the judging of candidates. After mapping transaction itemsets to a TN-tree, the itemsets’ exact utility values can be retrieved from the tree, so HUIL-TN and HUI-TN mine HUIs without generating candidates.

### 4.4 Algorithm analysis

**Property 1** (TN-tree Completeness). *Given a transaction dataset DB and a minimum utility value min*_*uti*, *its corresponding TN-tree contains the complete information of DB in relevance to HUI mining with the length k constraint*.

Based on the TN-tree construction process, all transactions itemsets that contains the same (local) promising items are mapped to one path (for example, *T*_4_ and *T*_7_ in Table 1 are mapped to one path in Fig 1), and have shared the same tail-node. The sum of utility of each item in those transactions are stored to the field *piu* on the tail-node. Thus, the utility of a high utility itemset *X*, whose length is not greater than *k*, can also be retrieved from the corresponding tail-nodes.

**Property 2**. *Let DB be a dataset*, *subDB be a sub dataset of itemset X*, *and itemset Y be in subDB and X* ∩ *Y* = ∅. *Then, the utility of X* ∪ *Y* *in DB is equivalent to the utility of X* ∪ *Y* *in subDB*, *and itemset X* ∪ *Y is a HUI if and only if it is a HUI in subDB*.

*Proof*. Based on the sub dataset construction process in Example 3 and Definition 15, all transactions containing itemset *X* ∪ *Y* are mapped to *subDB*. Thus, the utility of itemset *X* ∪ *Y* in *DB* is equivalent to the utility of *X* ∪ *Y* in *subDB*. So itemset *X* ∪ *Y* is a HUI in *DB* if and only if its utility in *subDB* is not less than the minimum utility value in *subDB*.

**Property 3** (HUIL-TN Correctness). *Given a base-itemset X, whose base utility is BU, for any remaining promising item Q in the subDB*, *(1) if RMU* < *min*_*util then any superset of itemset X* ∪ {*Q*} *is not a HUI; (2) if BU* + *NU* ≥ *min*_*util then itemset X* ∪ {*Q*} *is a HUI, otherwise not a HUI*.

*Proof*. (1) Firstly, based on the (sub) header table construction process, the *MU* value in a (sub) header table includes the utility values of (local) unpromising items in the corresponding transactions. Secondly, after an item of a (sub) header table is processed, algorithm HUIL-TN have mined all HUIs containing this item. So this algorithm needs not consider those processed items when it processes the remaining items of a (sub) header table. Based on these two reasons mentioned above, we need re-calculated the *MU* value of an item in a (sub) header table. In algorithm HUIL-TN, the *RMU* value is the new *MU* value of itemset *X* ∪ {*Q*} and it does not include the utility values of the two kinds of items mentioned above (unpromising items and processed items). According to Theorem 1, any superset *Y* of itemset *X* ∪ {*Q*} (*Y* does not include unpromising items and the processed items in sub header and its length is not greater than *k*) is not a HUI if *RMU* is less than the minimum utility value.

(2) Let *subDB* be the sub dataset of itemset *X* (if *X* is null, *subDB* is the original dataset). Based on the sub TN-tree construction process, the value $\sum_{i=1}^{k}\left(N_{i}.bu+N_{i}.nu\right)$ is the utility of itemset *X* ∪ {*Q*} in *subDB*. According to Property 2, itemset *X* ∪ {*Q*} is a high utility itemset if and only if $\sum_{i=1}^{k}\left(N_{i}.bu+N_{i}.nu\right)$ is not less than the minimum utility value.

Property 3 guarantees all itemsets mined by algorithm HUIL-TN are HUIs. For example, in Example 3, the utility value of each new base-itemset (*BU*+ *NU*) is obtained from the tree, so it is a HUI if its utility value is not less than the minimum utility value. Note that in the special case of *X* is null, a sub TN-tree is a global TN-tree.

## 5 Experimental results

We evaluated the performance of the proposed algorithms on eight standard datasets. Table 3 shows the characteristics of these transaction datasets, where column (I) shows the number of distinct items, column (AS) shows the average size of transactions, column (T) shows the total number of transactions, and the last column (DS) shows the percentage of total distinct items that appear in each transaction. The last column (DS) in Table 3 provides a measure of whether a dataset is dense or sparse. In general, a sparse dataset contains fewer items per transaction, but the set of items is relatively large. A dense dataset, in contrast, has many items per transaction, but the set of items is relatively small. Therefore, when the value of DS parameter of a dataset is relatively low (e.g., less than or equal to 10.0), a dataset is said to be sparse [37]. For example, the datasets Chess, Mushroom, Connect and Accident are dense datasets, and the other four datasets are sparse datasets. These datasets can be downloaded from the website [38]: http://www.philippe-fournier-viger.com/spmf/.

**Table 3:** Dataset characteristics.

Dataset	I	AS	T	DS
Chess	76	37	3,196	48.68%
Mushroom	119	23	8,124	19.33%
Connect	129	43	67,557	33.33%
Accident	468	33.8	340,183	7.22%
Pumsb	2,111	74	49,046	3.50%
BMS	497	4.8	59,601	0.96%
Retail	16,470	10.3	88,162	0.0625%
Chainstore	46,086	7.2	1,112,949	0.0156%

We compare the performance of algorithms HUIL-TN and HUI-TN with four state-of-the-art algorithms, namely EFIM [7], D2HUP [8], HMiner [6] and ULBMiner [9]. All algorithms were written in Java programming language. The source code of four compared algorithms can be downloaded from the website [38]: http://www.philippe-fournier-viger.com/spmf/.

The configuration of the testing platform is as follows: Windows 10 operating system, 16G Memory, Intel(R) Core(TM) i5-4460 CPU @ 3.20 GHz.

In order to assess the performance of the proposed algorithms, the runtime, memory usage and scalability were tested in different situations.

### 5.1 Runtime performance comparison

**Fig 7:**
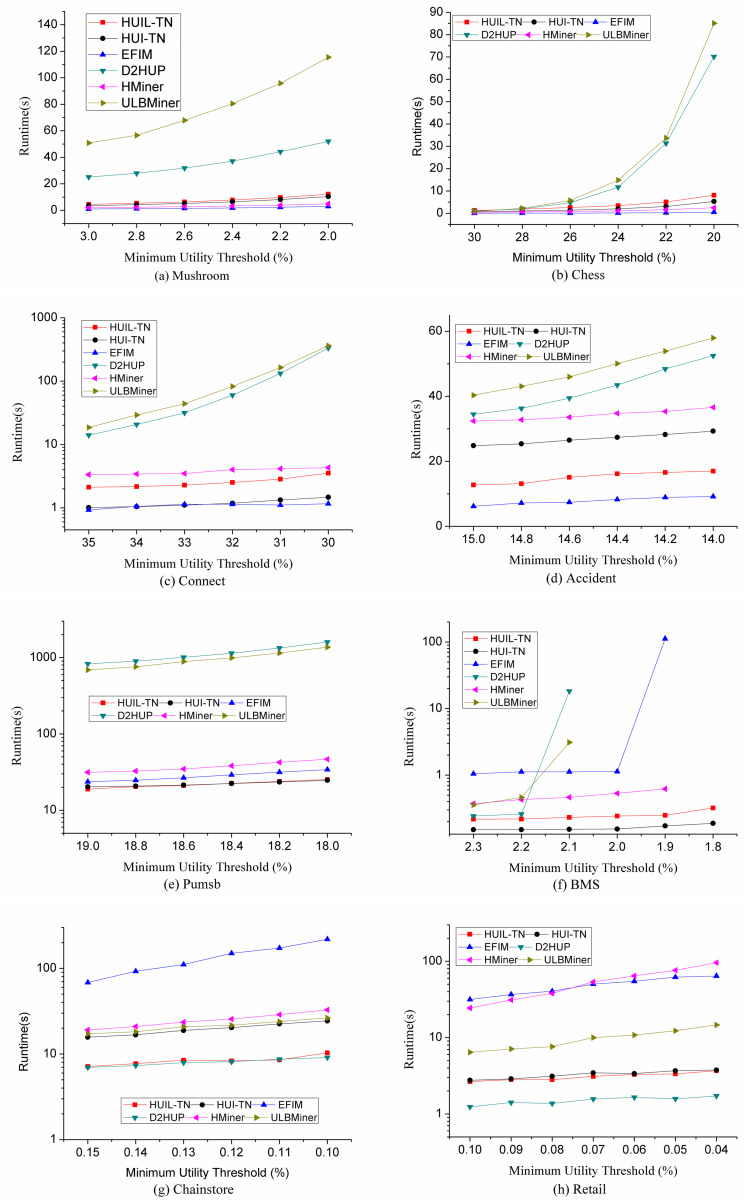
Comparison of runtime.

The running time of six algorithms is compared as shown in Fig 7, and the resulting HUIs mined by all algorithms are identical. Fig 7 shows the comparison of running time on each dataset under various minimum utility thresholds. The smaller the minimum utility threshold (*η*), the longer the algorithm will take. On datasets Chainstore and BMS, when the minimum utility threshold is too small, ULBMiner, D2HUP, EFIM, and HMiner would cause memory overflow or run too much time (more than 1 hour), so the corresponding data points are omitted in Fig 7.

It can be seen from Fig 7 that the performance of algorithms HUIL-TN and HUI-TN is stable on dense and sparse datasets. Algorithms HUIL-TN and HUI-TN outperform the other four algorithms on datasets Pumsb, BMS and Chainstore. On the other four datasets, the performance of HUIL-TN and HUI-TN also reach to the best. For example, on BMS, the execution times of HUIL-TN and HUI-TN are less than 1 second when the minimum utility threshold is 2.3%. Fig 7 also shows that HUIL-TN and HUI-TN not only outperform significantly in terms of time efficiency, but also develop more smoothly when the threshold decreases.

The reason that HUIL-TN and HUI-TN work well in terms of runtime is as follows.

1. These two algorithms map transaction itemsets to a TN-tree, and exact utility values of any existing itemsets in the dataset can be retrieved from the tree. Thus, it can find all HUIs from the tree using the pattern-growth approach.
2. These two algorithms use the *RMU* or *RTWU* values to determine whether a tree should be generated. If they generate less trees and process less itemsets during mining process, the performance of these two algorithms have been improved in terms of runtime and memory.

### 5.2 Memory performance comparison

**Fig 8:**
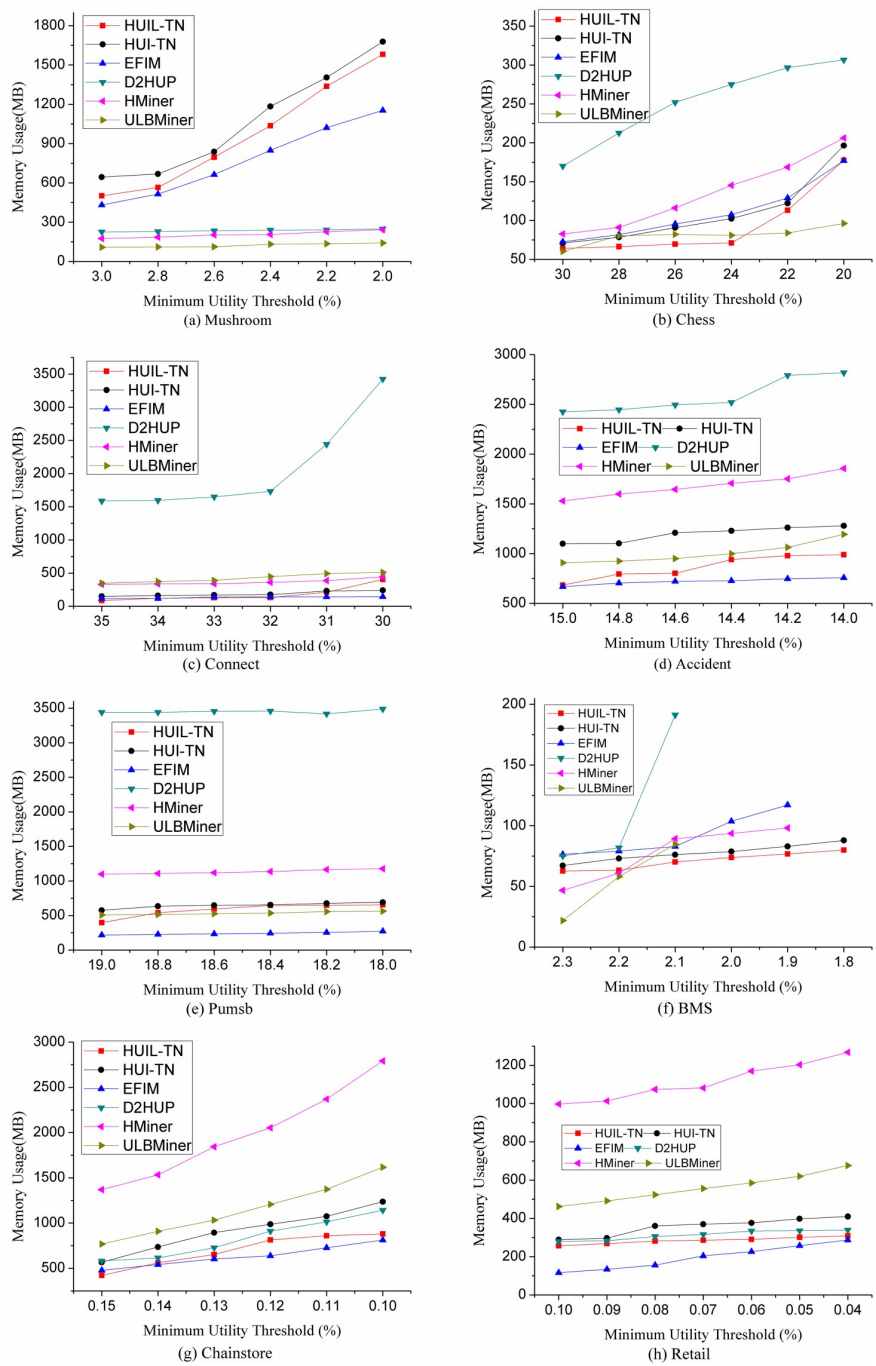
Comparison of memory usage.

In this section, we compare memory usage of six algorithms in different situations. The memory usage is tested under the same experimental conditions as those of the runtime tests in the above section. The experimental results are shown in Fig 8. From Fig 8, we can find that HUIL-TN and HUI-TN consume fewer memories on seven datasets Chess, Connect, Accident, Pumsb, BMS, Chainstore, and Retail. One reason is that the proposed algorithms HUIL-TN and HUI-TN can map transaction dataset to a tree. The other reason is that the proposed algorithms use the *RMU* or *RTWU* value to identify the candidate, so fewer sub-trees are created and less space overhead is needed. But on the dataset Mushroom, the proposed algorithms costs more space. The main reason is that this dataset can generate too many HUIs, i.e. 977,990 HUIs with *η* = 2.8%.

### 5.3 Sclalability experiments

**Fig 9:**
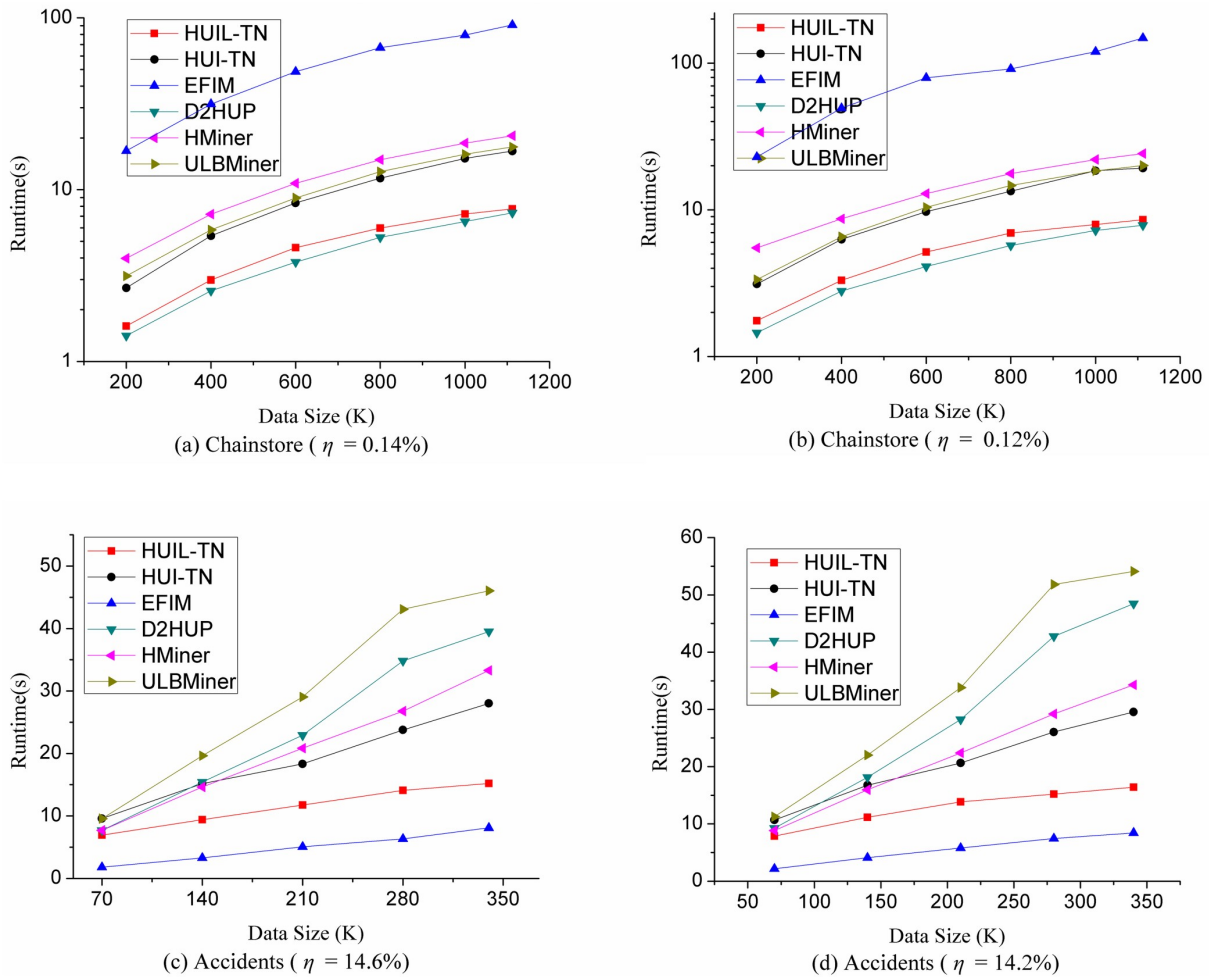
Comparison of runtime under varied dataset size.

**Fig 10:**
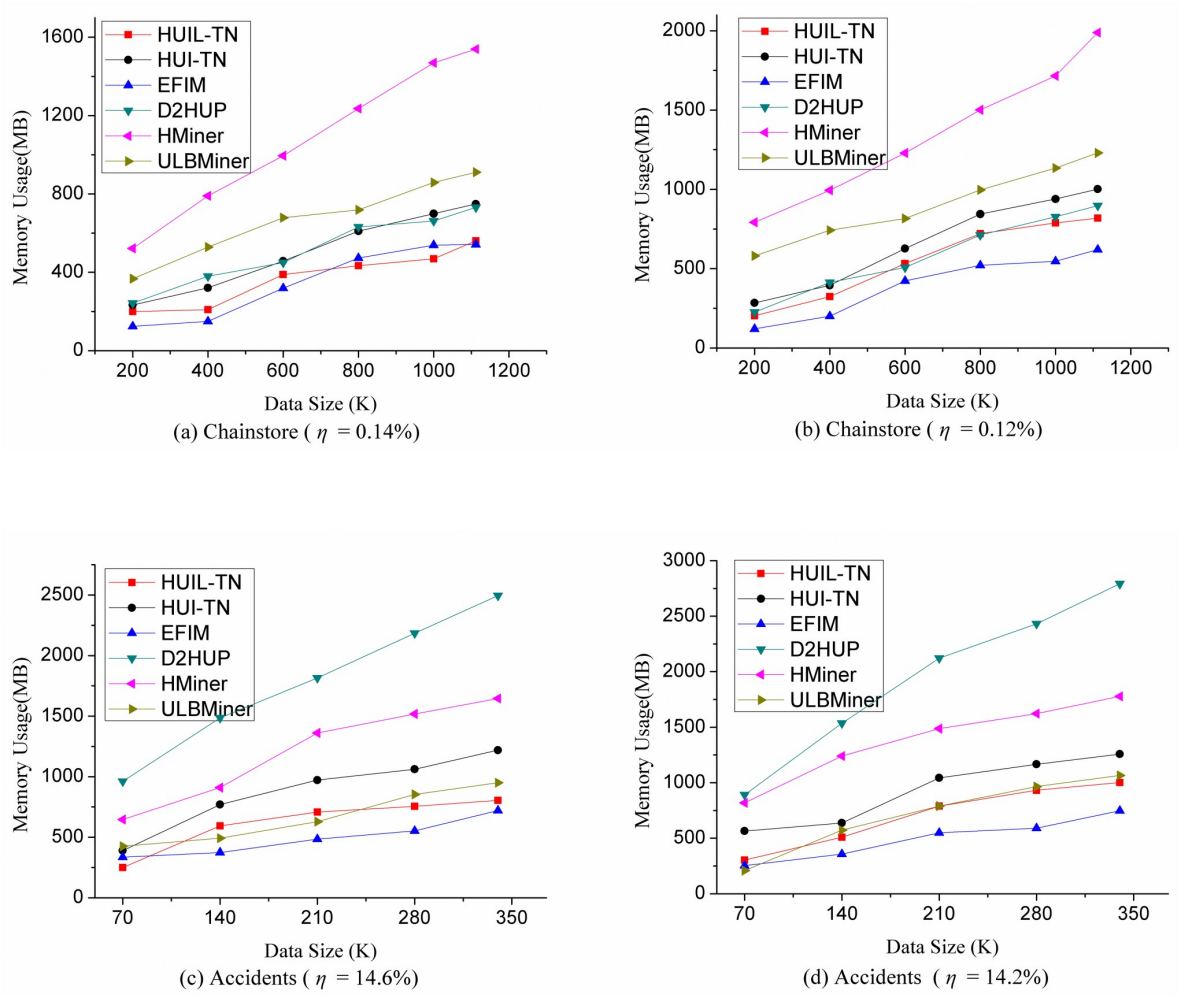
Comparison of memory usage under varied dataset size.

In order to test the scalability of the proposed algorithms, we choose two big datasets Chainstore and Accidents, varied the size of these two datasets, and assess the performance of runtime and memory usage. Chainstore is a sparse big data, and Accident is a dense big dataset. The experimental results are shown in Figs 9 and 10. The more transactions processed, the more time and memory it takes for mining HUIs. It can be seen from Figs 9 and 10 that HUIL-TN and HUI-TN cost less time and memory under different situations, and the performance of HUIL-TN and HUI-TN is stable on sparse and dense datasets.

In summary, the algorithms HUIL-TN and HUI-TN can map the dataset to a tree, and directly obtain utility value of an itemset from the tree. These two algorithms can directly obtain HUIs from the tree using the pattern-growth approach. As a result, the performance of these two proposed algorithms has been improved in terms of runtime, and is stable in different situations.

## 6 Conclusions

In this paper, we proposed two efficient algorithms, called HUIL-TN and HUI-TN, for mining HUIs from transaction dataset. Using the pattern-growth approach, it can mine HUIs directly from a TN-tree without generating candidate itemsets through only two scans of a dataset. A novel data structure TN-tree was proposed for storing the transaction dataset. The utility value of each item of an existing itemset of a dataset is stored in a tail-node. Thus, HUIL-TN and HUI-TN can retrieve the utility value of the itemset from the tree, i.e., find HUIs from this tree without using over-esitimated utility value. Moreover, HUIL-TN reduces the estimated utility value of an itemset by using the *RMU* value; as a result, it reduces the number of items in header table and enhances computing efficiency. In the experiments, dense datasets, sparse datasets, real-life datasets, and datasets containing many long transaction itemsets are used to evaluate the performance of our algorithms. Experimental results showed that our algorithms exceed or close to the best performance on all datasets in terms of running time, while other algorithms can only excel in certain types of dataset. Scalability tests were also performed and our algorithms obtained the flattest curves among all competitors.
